# Advanced Liver Fibrosis Is Independently Associated with Palmitic Acid and Insulin Levels in Patients with Non-Alcoholic Fatty Liver Disease

**DOI:** 10.3390/nu10111586

**Published:** 2018-10-29

**Authors:** Kátia Cansanção, Luana Silva Monteiro, Nathalie Carvalho Leite, Alberto Dávalos, Maria das Graças Tavares do Carmo, Wilza Arantes Ferreira Peres

**Affiliations:** 1 Institute of Nutrition Josué de Castro of Federal University of Rio de Janeiro (UFRJ), Rio de Janeiro 21.941-902, Brazil; kcansancao@gmail.com (K.C.); tcarmo@nutricao.ufrj.br (M.d.G.T.d.C.); 2 Nutrition Course, Federal University of Rio de Janeiro (Campus UFRJ-Macaé), Macaé, Rio de Janeiro 27.930-560, Brazil; luananutrir@gmail.com; 3 Department of Internal Medicine, University Hospital Clementino Fraga Filho, School of Medicine of UFRJ, Rio de Janeiro 21.941-902, Brazil; nathaliecleite@gmail.com; 4 Laboratory of Epigenetics of Lipid Metabolism, Madrid Institute for Advanced Studies (IMDEA)-Food, CEI UAM+CSIC, 28049 Madrid, Spain; alberto.davalos@imdea.org

**Keywords:** non-alcoholic fatty liver disease, liver fibrosis, fatty acid composition, dietary lipids intake, nutritional assessment

## Abstract

Changes in lipid metabolism occur during the development and progression non-alcoholic fatty liver disease (NAFLD). However, the fatty acid (FA) profile in red blood cells (RBC) from patients with liver fibrosis remains unexplored. Thus, the goal of this study was to evaluate the fatty acid profile in RBC, dietary lipid intake and insulin resistance indicators in patients with NAFLD, according to the degree of hepatic fibrosis. Using elastography, patients were classified with (*n* = 52) and without (*n* = 37) advanced liver fibrosis. The fatty acid profile in RBC was analyzed using gas chromatography and the lipid intake was evaluated through a 24-h dietary recall. Subjects with advanced liver fibrosis had higher levels of palmitic, stearic and oleic acid and total monounsaturated fatty acid (MUFA) and insulin (*p* < 0.05), and lower levels of elongase very long chain fatty acids protein-6 and the delta-5-desaturase enzymatic activity (*p* < 0.05). These results suggest a lack of regulation of enzymes related to FA metabolism in patients with advanced fibrosis.

## 1. Introduction

Non-Alcoholic Fatty Liver Disease (NAFLD) is considered the most common cause of chronic liver disease and is the second main reason for liver transplant [1]. Liver fibrosis is the main predictor of progression of NAFLD. The gold standard for the assessment of hepatic necroinflammation and fibrosis is liver biopsy. However, the procedure offers some risks and is too invasive to be performed in some patients. To overcome these limitations, transient hepatic elastography, a noninvasive method to assess liver fibrosis, has been widely used [2,3].

Liver fat accumulation results from an imbalance between fatty acid (FA) uptake and disposal, which leads to an increase in oxidative stress, activating stellate cells. Stellate cell activation contributes to hepatic fibrosis and, consequently, to the development of non-alcoholic steatohepatitis (NASH), cirrhosis and hepatocellular carcinoma [4].

The pathogenesis of NAFLD is complex and multifactorial [4]. The accumulation of an excess of lipids in the liver, mainly free fatty acids and triglycerides, has been attributed, at least in part, to insulin resistance [5]. Insulin promotes de novo lipogenesis (DNL), which increases hepatic synthesis of palmitic acid and consequently the risk of lipotoxicity, resulting in hepatocyte apoptosis and NAFLD progression [6,7]. In addition to insulin resistance, a diet high in saturated fat has also been described as an independent risk factor for the development of NAFLD [8]. Thus, diet, elongation and desaturation enzymatic processes related to lipid metabolism, as well as DNL, can influence not only FA composition in the liver, but also in red blood cells (RBC) [9,10]. Thus, the FA composition in RBC can be used as a good and noninvasive marker of lipid metabolism in an injured liver [10,11]. Studies demonstrate changes in lipid metabolism during the development of hepatic steatosis and progression to steatohepatitis, however, the composition of fatty acids in the individuals with and without liver advanced fibrosisremains unexplored [9,11]. Therefore, the aim of this study was to evaluate the fatty acid profile in RBC, dietary lipid intake and insulin resistance indicators in patients with non-alcoholic fatty liver disease (NAFLD), according to the degree of hepatic fibrosis.

## 2. Materials and Methods

### 2.1. Study Design and Participants

This cross-sectional study took place between January 2014 and January 2015. It included patients with NAFLD diagnosis confirmed by ultrasound and the patients with liver fibrosis quantified by transient hepatic elastography. Individuals with viral hepatitis, cancer, gastrointestinal tract inflammatory diseases, fever, or infectious focus were excluded from the study. Other exclusion criteria were the use of steroid or non-steroid anti-inflammatories, immunomodulation agents, or antibiotics, transplant, trauma, surgery, or hospital stay in the last 30 days, >20 g/day alcohol consumption, pregnancy, and consumption of fish oil supplements in the last 6 months.

The present study was conducted according to the guidelines of the Declaration of Helsinki and all procedures involving human subjects/patients were approved by the Research Ethics Committee of the University Hospital, registration number 080434/2013. All subjects/patients provided written informed consent.

### 2.2. Transient Hepatic Elastography and Advanced Fibrosis Definition

To evaluate liver stiffness, all the patients were subjected to FibroScan^®^ elastography (model 502, Echosens, Paris, France) by a trained examiner, always employing first an M probe. The XL probe was used if the examination was not possible or not reliable with the M probe. The median value represented the liver elastic modulus. Only results with 10 valid measurements, over 60% success percentage and interquartile range (IQR)/average stiffness below 30% were included. Liver stiffness was expressed in kilopascal (kPa). The success rate was calculated as the number of successful measurements divided by the total number of measurements. Two groups were identified according to the liver fibrosis median: without advanced fibrosis < 7.9 kPa and with advanced fibrosis ≥ 7.9 kPa. This was based on a study [12] that proposed the optimum cut-off point of 7.9 kPa for the advanced fibrosis diagnosis in patients with NAFLD.

### 2.3. Biochemical Evaluation

Blood samples were obtained after a 12-h fast and then centrifuged for plasma and serum separation. To obtain the RBC, 3 washes were performed with isotonic sodium chloride (NaCl 0.9%, *w*/*v*) discarding the supernatant at each stage. After the third wash the RBC were aliquoted to an eppendorf and sodium dithionite (1.0% *w*/*v*) was added to preserve the double bonds of the unsaturated fatty acids [13]. Samples were maintained at −80 °C until analysis. Additional examinations were performed using commercial kits as part of routine HUCFF service [total cholesterol, low-density lipoprotein (LDL-c) and high-density lipoprotein (HDL-cholesterol) cholesterol, triglycerides, gamma-glutamyl transferase (GGT), alkaline phosphatase (ALP), aspartate transaminase (AST), alanine transaminase (ALT), glycated hemoglobin and glucose]. In addition, the non-esterified fatty acids (NEFA) and fasting plasma insulin were analyzed by the electrochemiluminescence and spectrophotometry methods, respectively, by the clinical analysis laboratory of the Faculty of Pharmacy of the Federal University of Rio de Janeiro. The Homeostatic Model of Assessment-Insulin Resistance (HOMA-IR) index was obtained using the formula: HOMA-IR = insulin (mU/L) × glucose (mmol/L)/22.5.

### 2.4. Assessment of Fatty Acid Composition of Red Blood Cells (RBC)

To obtain the fatty acids, the Lepage and Roy [14] methodology was used with the addition of the internal standard tritridecanoic acid (13:0), (Sigma-Aldrich, Saint Louis, MO, USA), in stored RBC aliquots. Subsequently, a solution of chloroform methanol (2:1) was added for extraction and purification of the lipids. The total lipid extracts were saponified and adding 2 mL of 4:1 (*v*/*v*) methanol: hexane solution and 100 μL of acetyl chloride in the cold the fatty acids were methylated. The samples were kept at room temperature for 24 h for transesterification [15].

After the 24 h period the samples were analyzed using a GC 7890A chromatograph equipped with a hydrogen flame ionization detector and EZChrom Elite CDS software (Agilent Technologies, Inc., Santa Clara, CA, USA). The separation of the FAs was performed on a capillary column SP 2560 (bisulopropyl polysiloxane, 100 m × 0.25 mm ID, thickness of 0.20 μm; Supelco, Bellefonte, PA, USA). Chromatographic conditions were similar to those described by de Assumpção et al. [16]. The identification of the fatty acid methyl esters was accomplished by comparing the relative retention time with authentic standards (Reference Standard GLC 463, Nu-Chek Prep, Inc., Elysian, MN, USA), the results were expressed as% by weight of total fatty acid.

### 2.5. Fatty Acid Indexes and Estimation of Enzymatic Activities

Enzymatic activities were estimated as product-to-precursor ratios of individual FAs in RBC, as follows: elongation of very long chain fatty acids protein 6 (ELOVL6) activity as the ratio of 18:0/16:0; stearoyl-CoA desaturase 1 (SCD1) activity as the ratio of 16:1n-7/16:0 and 18:1n-9/18:0; delta-5-desaturase (D5D) as the ratio of 20:4n-6/20:3n-6; and delta-6-desaturase (D6D) as the ratio of 18:3n-6/18:2n-6. Additionally, the 16:0/18:2n-6 ratio was used as an index of DNL and the omega 3 index 20:5(n-3) + 22:6(n-3) was also included.

### 2.6. Nutritional Evaluation

Weight and height of the participants was measured on an upright scale (PL 180, Filizola SA, São Paulo, SP, Brazil). The body mass index (BMI) was calculated by dividing weight (kg) by height (m) squared.

The waist circumference (WC) was evaluated encircling the midpoint between the last rib and the iliac crest [17]. The adequacy of WC was assessed according to the cut-off point of the International Diabetes Federation (IDF) [18]. For the hips perimeter, the gluteus maximus region with the largest circumference was measured [17]. The waist/height ratio (WHtR) was waist perimeter (m) divided by the height (m) [19]. The body adiposity index (BAI) [20] was calculated using the formula: BAI = ((hip circumference)/(height)^1.5^) − 18)) =. The body shape index (ABSI) [21] was obtained by the following equation: (ABSI) = WC (cm)/BMI^2/3^ × Height(m)^1/2^.

### 2.7. Evaluation of Lipid Ingestion

The dietary ingestion of lipids was estimated for three non-consecutive days by 24 h-recall (R24h), in which the person informed all the food and beverages ingested during the last 24 h prior to the interview, as well as the preparation method and the quantity ingested in household measurements. A specific data entry program was used. It was composed of approximately 1500 items (food and beverages), which were selected from 5686 records in the food and beverage database of the 2002–2003 *Programa de Orçamento Familiar* (*POF*—Household Budget Program) of the Brazilian Institute of Geography and Statistics—IBGE. To estimate lipid intake, the tables of nutritional composition and household measures were compiled specifically to analyze the foods and preparations mentioned 2008–2009 POF [22]. The results of the nutritional composition related to each of the three R24h were adjusted, to correct for intrapersonal variability, using the Multiple Source Method (https://msm.dife.de) [23]. The classification of nutrients adequacy was performed using the reference values proposed by the NCEP ATP III (Third Report of the National Cholesterol Education Program Adult Treatment Panel III) [24,25].

### 2.8. Statistical Analysis

Data analysis was performed using the Statistical Package for the Social Sciences (SPSS) software, version 21.0. Non-parametric methods were applied, since the variables did not display normal distribution (Gaussian), due to the rejection of the normality hypothesis of the Shapiro-Wilk test. The data were expressed in median and interquartile range (IQR) for numerical variables and in frequency (*n*) and percentage (%) for categorical data. The Mann-Whitney test was used to compare the anthropometric, biochemical, and fatty acid composition parameters between the advanced and non-advanced fibrosis groups. Analysis of association between categorical data was performed using the *χ^2^* test. Additionally, binary logistic regression was used to identify the association between fibrosis status and FA composition, BMI, fasting insulin, HOMA-IR, glucose, sex and age. Adjusted odd ratios were applied to the predicted probability of association between factors with 95% confidential intervals. The indices, including age, BMI, sex, insulin, glucose and HOMA-IR, were adjusted as covariates in the logistic regression model. In the univariate analysis, only the variables with *p*-value lower or equal to 0.20 were included. In the final model, all the variables with *p* < 0.05 were included. The criteria adopted for determining significance was 5%.

## 3. Results

### 3.1. Characteristics of the Participants

The general characteristics of the participants are shown in Table 1. Among the 89 patients with NAFLD diagnosis included, 58.43% had advanced fibrosis. The average age of the patients was 61.53 ± 9.92 years, varying between 26 and 84 years, and 66 (74.2%) were female. Subjects with advanced fibrosis had higher concentrations of fasting insulin, ALT, AST, GGT and BMI than those without advanced fibrosis (all *p* < 0.05). Type 2 diabetes was also more frequent among patients with advanced fibrosis (*p* = 0.045) (Table 1). All evaluated patients (100%) presented high waist circumference, according to the IDF.

### 3.2. Fatty Acid Profile of RBC

Table 2 presents the content of each fatty acid, as a percentage of the total fatty acids, in the RBC. Patients with advanced fibrosis had higher percentages of palmitic acid (16:0), stearic acid (18:0), oleic acid (18:1n-9) and total monounsaturated fatty acids (MUFA) than patients without advanced fibrosis (*p* < 0.001; *p* = 0.027; *p* = 0.004; *p* = 0.015, respectively). The analysis of enzymatic activities based on RBC FA composition revealed that both D5D activity (ratio of 20:4n-6/20:3n-6) and ELOVL6 activity (ratio of 18:0/16:0) were lower in patients with advanced fibrosis than in those without advanced fibrosis (*p* = 0.010; *p* = 0.014, respectively).

### 3.3. Assessment of Dietary Lipid Intake

All patients (100%) reported dietary intake of monounsaturated fat below the recommended levels, and 97.8% reported elevated consumption of saturated fat (Table 3). However, lipid intake was similar for patients with and without fibrosis (Table 4).

### 3.4. Factors Associated to Liver Fibrosis

The percentage of palmitic acid (16:0) and fasting insulin associated significantly with a greater odds ratio for advanced fibrosis in the binary logistic regression, adjusted for age, sex, BMI, sex, insulin, glucose and HOMA-IR (Table 5).

## 4. Discussion

In this study with NAFLD patients, advanced liver fibrosis, a marker of liver disease progression, was significantly associated with a higher percentage of palmitic acid (16:0), stearic acid (18:0), oleic acid (18:1n-9) and total MUFA in their RBC. Furthermore, patients with advanced fibrosis had lower D5D activity (ratio of 20:4n-6/20:3n-6) and ELOVL6 activity (ratio of 18:0/16:0). Finally, advanced fibrosis was independently associated with fasting insulin, and with a high percentage of palmitic acid in RBC, in a binary logistic regression. Thus, in this study, we describe for the first time an association between the profile of fatty acids in RBC and hepatic fibrosis in NAFLD patients.

The lower 18:0/16:0 ratio in patients with advanced fibrosis could result from increased levels of palmitic acid in their RBC, as this ratio reflects the activity of the ELOVL6 enzyme, which participates in the elongation of palmitic acid (16:0) to stearic acid (18:0). Similar findings were described in a study of hepatic tissue in patients with steatosis and NASH, where the ratio of 18:0/16:0 was found to be lower in NASH. In fact, this is the most important factor related to the score of liver steatosis [26]. According to this and our data, the reduction of the 18:0/16:0 ratio occurs both in patients with strong accumulation of fat in the liver and in patients with advanced liver fibrosis, suggesting that the reduction of this ratio is indicative of more advanced NAFLD. Moreover, according to our findings, palmitic acid was the only FA pointed out as an independent factor associated with the occurrence of advanced fibrosis. Indeed, studies indicate that palmitic acid (16:0) is the most cytotoxic fatty acid for the liver, leading to cell injury and death [27]. Palmitic acid has been shown to activate hepatic stellate cells, through inflammasomes and hedgehog signaling, leading to the progression of hepatic fibrosis [28].

In our study, the proportion of stearic acid (18:0) in RBC was also significantly increased in patients with advanced fibrosis. This suggests that stearic acid also has a significant cytotoxic potential, contributing to the progression of fibrosis [29]. Additionally, we found a significant increase in oleic acid and in total MUFA in the RBC of patients with advanced fibrosis. MUFA, as well as saturated fatty acids (SFA), may have a weak correlation with diet, as their metabolites depend not only on dietary intake. Besides, all the patients studied presented MUFA intake below maximum recommended values, suggesting that the increase observed in MUFA may be due to the rise in the endogenous production of oleic acid, which is the main substrate for hepatic synthesis of triglycerides [30].

The fatty acid composition of RBC can be influenced by the ingestion of lipids, as well as by their endogenous metabolism, through the desaturase enzymes that catalyze the formation of unsaturated fatty acids [9]. Here, we examined the influence of dietary intake on the fatty acid RBC profile. We observed that patients with advanced fibrosis did not present a higher intake of SFA compared to patients without advanced fibrosis, which suggests that the palmitic acid increase found in RBC reflects increased de novo lipogenesis (DNL), in addition to reduced ELOVL6 activity. This hypothesis is supported by another study that demonstrated that the amount of SFA in RBC was not associated with dietary intake [31]. In fact, a previous study reported that the percentage of palmitic acid in RBC does not depend on dietary intake, as it can also be synthesized endogenously. Indeed, this SFA is considered the main product of DNL [9].

We also observed a significant reduction in the estimated activity of the D5D enzyme in patients with advanced fibrosis, although no difference was found in the percentage of docosahexaenoic acid and eicosapentaenoic acid in RBC. Our findings corroborate a recent study [32] with NAFLD patients diagnosed by liver biopsy, where the estimated activity of D5D was significantly lower in NASH patients compared to healthy liver. Reduced D5D activity has been associated with an increased risk of type 2 diabetes mellitus and increased plasma triglycerides [32,33,34].

It is important to note that 100% of the evaluated patients presented high abdominal circumference according to the IDF. Furthermore, patients with advanced fibrosis had higher body and abdominal adiposity, measured by BMI and WHtR, respectively. These alterations can contribute to an increase of the flow of non-esterified fatty acids to the liver [35].

In this study, we did not use liver biopsy for fibrosis assessment, despite this being considered the gold standard for NAFLD spectrum conditions. Liver biopsy is an invasive procedure, which may lead to complications. Moreover, unequal distribution of fibrosis in the hepatic parenchyma may lead to false interpretation of the diagnosis and incorrect staging.

The self-reported 24-h dietary intake used in the study is a validated method of dietary assessment [36]. However, it presents some limitations, such as an incorrect estimation of the portion consumed and the reliance on the memory of the patient.

## 5. Conclusions

To our knowledge, this is the first study that evaluates the composition of fatty acids in RBC and lipid intake in patients with and without liver advanced fibrosis, assessed by transient hepatic elastography. Patients with advanced fibrosis presented higher percentage of the palmitic and stearic SFAs and of oleic MUFA in RBC, compared to patients without advanced fibrosis. As dietary intake of these fatty acids was similar in both groups, these changes may be associated with an increase in de novo lipogenesis, which is involved in the pathogenesis of NAFLD.Therefore, monitoring the intake of these nutrients may be important, together with lifestyle changes, to control de progression of NAFLD.

## Figures and Tables

**Table 1:** Characteristics of the study population.

Variables	Patients without Advanced Fibrosis (^#^LS Value < 7.9 kPa) *n* = 37		Patients with Advanced Fibrosis (^#^LS Value ≥ 7.9 kPa) *n* = 52		
	%		%		*p* value
Gender (F/M)	70.28/29.72		76.92/23.08		0.482
Hypertension	86.48		78.84		0.358
Type 2 diabetes	56.75		76.92		0.045
Hypertriglyceridemia	29.72		30.76		0.917
MS	75.67		78.84		0.725
	Median	IQR	Median	IQR	*p* value
Age, yr	62.00	57.00–68.00	62.50	55.00–67.75	0.508
BMI (kg/m²)	29.60	27.59–34.02	33.26	29.57–36.50	0.018 *
WC (cm)	105.00	97.75–110.00	109.00	98.62–116.75	0.116
WHR	1.00	0.93–1.06	0.99	0.94–1.07	0.708
BAI	31.69	29.00–37.11	35.35	29.32–38.82	0.240
WHtR	0.63	0.59–0.70	0.68	0.60–0.73	0.099
ABSI	0.0841	0.0810–0.0878	0.0834	0.0774–0.0888	0.580
ALT (U/L)	44.00	33.00–68.50	54.50	39.25–84.00	0.021 *
AST (U/L)	26.00	20.00–35.50	38.00	28.50–55.75	<0.001 *
Variables	Patients without advanced fibrosis (^#^LS value < 7.9 kPa) *n* = 37		Patients with advanced fibrosis (^#^LS value ≥ 7.9 kPa) *n* = 52		
	Median	IQR	Median	IQR	*p* value
GGT (U/L)	39.00	28.00–73.50	87.59	57.75–143.75	<0.001 *
ALP (U/L)	91.00	72.25–112.75	99.00	76.25–123.00	0.313
Serum insulin	14.90	8.45–22.75	20.05	12.32–28.05	0.044 *
HOMA-IR	4.08	2.38–5.90	4.56	3.44–8.20	0.113
NEFA (mcU/mL)	645.79	403.24–940.89	688.56	547.35–789.07	0.611
Total cholesterol (mg/dL)	170.00	151.50–218.50	185.00	152.25–214.75	0.727
LDL (mg/dL)	96.00	75.00–133.50	102.50	82.00–130.25	0.546
HDL (mg/dL)	46.00	37.50–54.50	42.00	35.25–51.75	0.272
Triglycerides (mg/dL)	133.00	81.50–229.50	151.50	99.50–247.50	0.410

Values are medians, interquartile ranges (IQRs), or % of patients. * Different between patients with and without advanced fibrosis, *p <* 0.05. Mann-Whitney test was applied to numerical variables and the chi-square (X^2^) test for categorical variables. ^#^LS, liver stiffness; MS, metabolic syndrome; BMI, body mass index; WC, waist circumference; WHR, waist/hips ratio; BAI, body adiposity index; WHtR, waist/height ratio; ABSI, body shape index; ALT, alanine transaminase; AST, aspartate transaminase; AST/ALT ratio, AST/ALT; GGT, gamma-glutamyl transferase; ALP, alkaline phosphatase; NEFA, non-esterified fatty acids; HOMA-IR, Homeostasis Model Assessment Method; HbA1c, Glycated hemoglobin; LDL, low-density lipoprotein; HDL, high-density lipoprotein.

**Table 2:** Lipid composition of red blood cells (% of total fatty acids).

Variables	Patients without Advanced Fibrosis (^#^LS Value < 7.9 kPa) *n* = 37		Patients with Advanced Fibrosis (^#^LS Value ≥ 7.9 kPa) *n* = 52		
FA	Median	IQR	Median	IQR	*p* value
16:0	19.40	17.91–21.49	21.89	19.62–23.32	0.001 *
18:0	16.73	15.83–18.21	17.89	16.85–18.81	0.027 *
Ʃ SFA	48.63	44.11–54.36	46.48	44.50–50.11	0.141
16:1(n-7)	0.78	0.49–1.27	0.77	0.39–1.88	0.882
18:1(n-9)	10.45	9.35–11.10	11.34	10.23–12.58	0.004 *
Ʃ MUFA	15.39	13.98–17.66	17.11	14.79–19.37	0.015 *
18:2(n-6)	8.06	7.08–9.31	8.40	7.48–10.11	0.202
20:4(n-6)	12.71	11.14–14.47	13.51	11.27–14.96	0.480
Ʃ n-6 PUFA	28.74	24.41–32.11	28.41	25.35–31.53	0.835
18:3(n-3)	0.40	0.30–0.68	0.38	0.20–0.57	0.424
20:5(n-3)	1.19	0.99–1.50	1.19	0.97–1.40	0.335
22:6(n-3)	3.43	2.69–4.12	3.07	2.52–3.85	0.324
Ʃ n-3 PUFA	6.68	5.84–7.72	6.53	5.69–7.55	0.674
Ʃ PUFA	35.82	31.28–39.84	35.20	32.93–37.85	0.790
(n-6):(n-3)	4.31	3.84–4.77	4.46	3.81–5.27	0.415
Variables	Patients without advanced fibrosis (^#^LS value < 7.9 kPa) *n* = 37		Patients with advanced fibrosis (^#^LS value ≥ 7.9 kPa) *n* = 52		
	Median	IQR	Median	IQR	*p* value
Omega-3 index	6.60	3.87–5.57	4.34	3.54–5.02	0.162
18:3(n-6):18:2(n-6)	0.03	0.00–0.06	0.00	0.00–0.06	0.288
20:4(n-6):20:3(n-6)	9.59	7.08–13.52	7.90	4.79–9.55	0.010 *
18:0:16:0	0.85	0.79–0.91	0.82	0.75–0.85	0.014 *
16:1(n-7):16:0	0.04	0.03–0.07	0.05	0.03–0.15	0.139
18:1(n-9):18:0	0.61	0.54–0.66	0.63	0.57–0.70	0.095
16:0:18:2(n-6)	2.33	2.10–2.75	2.44	2.19–2.87	0.233

Values are medians, interquartile ranges (IQRs), or % of patients. * Different between patients with and without advanced fibrosis, *p <* 0.05 (Mann- Whitney U test). ^#^LS, liver stiffness; Omega-3 index, % 20:5(n-3) + % 22:6(n-3); FA, fatty acid; SFA, saturated fatty acid; MUFA, monounsaturated fatty acid; PUFA, polyunsaturated fatty acid.

**Table 3:** Dietary intake of fat in patients with NAFLD in comparison with NCEP ATP III recommended values.

Nutrients	Recommendations	Median	IQR	Lower to Recommended (%)	Over Recommended (%)
Total fat, % kcal	25–35% *	31.59	29.40–34.54	5.6	22.5
Saturated fat, % kcal	<7% ^#^	32.80	30.43–36.72	-	97.8
MUFA fat, % kcal	Up to 20% ^#^	11.03	10.11–12.16	100	-
PUFA fat, % kcal	Up to 10% ^#^	6.87	6.14–7.87	93.3	-
Cholesterol (mg)	<200 mg/d ^#^	166.30	165.47–167.11	100	-

Values are medians, interquartile ranges (IQRs), or % of patients. MUFA, monounsaturated; PUFA, polyunsaturated. * Acceptable macronutrient distribution range (%)-DRIs [24]. ^#^ NCEP ATP III (Third Report of the National Cholesterol Education Program Adult Treatment Panel III) [25].

**Table 4:** Dietary intake of patients with and without advanced fibrosis.

Variables	Patients without Advanced Fibrosis (^#^LS Value < 7.9 kPa) *n* = 37		Patients with Advanced Fibrosis (^#^LS Value ≥ 7.9 kPa) *n* = 52		
	Median	IQR	Median	IQR	*p* value
Total fat, % kcal	31.71	29.56–35.33	31.57	28.65–34.10	0.391
Saturated fat, % kcal	32.64	30.11–36.72	32.81	30.78–36.96	0.914
MUFA, % kcal	11.16	10.11–12.67	11.00	10.00–11.92	0.373
PUFA, % kcal	6.92	6.36–7.52	6.73	6.05–8.18	0.696
Cholesterol (mg)	166.29	165.60–167.39	166.31	165.41–166.95	0.419

Values are medians or interquartile ranges (IQRs). Different between patients with and without advanced fibrosis, *p* < 0.05 (Mann–Whitney U test). ^#^LS, liver stiffness; MUFA, monounsaturated fatty acid; PUFA, polyunsaturated fatty acid.

**Table 5:** Independent predictors of liver fibrosis.

Multivariate					
	Regression Coefficient	SE	OR	95% CI	*p* Value
Insulin	0.097	0.04	1.10	1.01–1.19	<0.001
16:0	0.392	0.13	1.48	1.13–1.93	<0.001

SE, standard error of the mean; OR, odds ratio; 95% CI, 95% confidence interval; SFA, saturated fatty acids.

## Abbreviations

ABSI	body shape index
ALP	alkaline phosphatase
ALT	alanine transaminase
AST	aspartate transaminase
BAI	body adiposity index
BMI	body mass index
DNL	de novo lipogenesis
D5D	delta-5-desaturase
D6D	delta-6-desaturase
ELOVL6	elongation of longchain fatty acids family member 6
GGT	gamma-glutamyl transferase
HOMA-IR	homeostatic model of assessment- insulin resistance
IQR	interquartile range
IR	insulin resistance
kPa	kilopascals
LS	liver stiffness
MUFA	monounsaturated fatty acids
NAFLD	nonalcoholic fatty liver disease
NASH	non-alcoholic steatohepatitis
POF	programa de orçamento familiar
RBC	red blood cells
R24h	24 h recordatory
SCD1	stearoyl-CoA desaturase 1
SFAs	saturated fatty acids
SREBP-1c	sterol regulatory element-binding protein-1c
WC	waist circumference
WHR	waist/hips ratio
WHtR	waist/height ratio
